# A Major QTL, Which Is Co-located with *cly1*, and Two Minor QTLs Are Associated with Glume Opening Angle in Barley (*Hordeum vulgare* L.)

**DOI:** 10.3389/fpls.2016.01585

**Published:** 2016-10-24

**Authors:** XinZhong Zhang, BaoJian Guo, GuoFang Lan, HongTao Li, Shen Lin, Jun Ma, Chao Lv, RuGen Xu

**Affiliations:** ^1^Jiangsu Key Laboratory of Crop Genetics and Physiology, Yangzhou UniversityYangzhou, China; ^2^Co-Innovation Center for Modern Production Technology of Grain Crops, Yangzhou UniversityYangzhou, China; ^3^Key Laboratory of Plant Functional Genomics of the Ministry of Education, Yangzhou UniversityYangzhou, China; ^4^Institute of Barley Research, Yangzhou UniversityYangzhou, China

**Keywords:** *Hordeum vulgare* L., glume opening angle, doubled haploid, QTL, chasmogamous

## Abstract

Cleistogamous and chasmogamous are two opposing phenomena for flowering in barley. Cleistogamy limits the rate of outcrossing, and increases the cost of producing hybrid barley seeds. Selecting chasmogamous lines with a large glume opening angle (GOA) is essential for the utilization of barley heterosis. In the current study, 247 DH lines derived from a cross between Yangnongpi7 and Yang0187 were used to identify and validate quantitative trait loci (QTLs) associated with the GOA in different environments using SSR markers. Three QTLs associated with barley GOA were mapped on chromosomes 2H and 7H. The major QTL *QGOA-2H-2* was mapped on chromosome 2H with the flanking markers of KDH and GBM1498, explaining 63.92% of the phenotypic variation. The marker KDH was developed from the gene *Cly1*, which was the candidate gene for *QGOA-2H-2*. This new marker can be used to identify barley chasmogamous lines with a large GOA. The two minor QTLs were validated at all three locations across two seasons after removing DH lines carrying the candidate gene *Cly1* of *QGOA-2H-2*.

## Introduction

For self-pollinating crops like barley and wheat, their cleistogamous flowers remain mechanically sealed throughout the entire flowering period. The closed morphology of cleistogamous flowers hinders them from exposing their reproductive organs and forces self-pollination to occur (Schemske, 1978; Culley and Klooster, 2007). The natural outcrossing rate of barley is approximately 1.7% (Parzies et al., 2000), which is too low for the commercial production of hybrid seeds. In contrast to cleistogamy, chasmogamy is a plant reproductive mechanism in which pollination occurs in chasmogamous flowers. Most chasmogamous flowers are cross-pollinated by biotic (e.g., insects) or abiotic (e.g., wind) agents with the rate of outcrossing determined by several floral traits including stigma and anther size, anther extrusion, aspect of flag leaf, glume opening angle (GOA), and flower opening duration (Taillebois and Guimaraes, 1986). Among these traits, GOA is the most important factor affecting the extent of outcrossing (Mahalingam et al., 2013). Therefore, research should be conducted to understand the genetic mechanism of barley GOA, which could provide important information for breeding of chasmogamous lines with a large GOA.

Inheritance of chasmogamic and cleistogamic glume has been studied in various crop species. Mutation has been one of the main tools for studying cleistogamic glume in rice. Recessive genes, *d7* and *ld(t)*, were reported to control rice cleistogamic glume (Nagao and Takahashi, 1963; Maeng et al., 2006). A cleistogamic gene *superwoman1 (SPW1)* was isolated and proved to have no influence on other agronomic traits (Yoshida et al., 2007). All these rice cleistogamic genes were dependent on the mutation sites of mutants. In wheat, chasmogamy was determined by a single dominant gene *Cl* (Chhabra and Sethi, 1991). Inheritance of chasmogamic and cleistogamic glume was more complex in barley. The segregation pattern observed in three barley F_2_ populations grown in multi-years at multi-locations indicated a single-gene inheritance of chasmogamy with complete dominance (Ceccarelli, 1978). In another study, cleistogamy was also reported to be controlled by a single recessive gene (Kurauchi et al., 1993). Using three different populations, *cly1* and *Cly2*, controlling cleistogamy, were mapped in the same region of chromosome 2HL, suggesting the possibility of pleiotropic effect or single effect of two tightly linked genes. Subsequently, the interval was reduced to 7 kb according to the synteny with rice chromosome 4 (Turuspekov et al., 2009). The cleistogamic gene *cly1* were positionally cloned (Nair et al., 2010). In addition, *HvAP2* was identified using both genome-wide association and bi-parental mapping, which determined the density of grains on the barley inflorescence (Houston et al., 2013). On the molecular level, *cly1*/*HvAP2* encodes a transcription factor containing two AP2 domains and a putative microRNA miR172 targeting site, which was an ortholog of *Arabidopsis thaliana* AP2 (Nair et al., 2010; Houston et al., 2013). All data indicated that this heterochronic change of miR172 targeting site was responsible for the striking differences in the size and shape of crop flowers (Nair et al., 2010; Houston et al., 2013; Ning et al., 2013).

Chasmogamy and cleistogamy were regarded as qualitative characters and measured by the size of lodicules (Nair et al., 2010) or the visibility of anthers (Turuspekov et al., 2004, 2009). Nevertheless, GOA has been shown to be a typical quantitative character (Uga et al., 2003; Lv et al., 2009; Lan et al., 2013). Using rice recombinant inbred lines, three quantitative trait loci (QTLs) for rice GOA were mapped on chromosomes 2, 8 and 9, respectively (Uga et al., 2003). Two or more genes were reported to be involved in wheat GOA and one of them was mapped on the short arm of chromosome 2B (Gilsinger et al., 2005). Although, inheritance analysis indicated that GOA was controlled by a major gene plus multiple minor genes (Lv et al., 2009), the specific alleles for GOA were still unknown. The aim of this study was to identify QTLs associated with barley GOA using a DH population derived from a cross between Yangnongpi7 and Yang0187.

## Materials and Methods

### Plant Materials

A total of 247 doubled haploid (DH) lines were derived from a cross between Yangnongpi7 and Yang0187 using anther culture method. Both Yangnongpi7 and Yang0187 are two-rowed barley varieties bred by Yangzhou University. Yangnongpi7 is a glume closing variety but Yang0187 is a glume opening variety.

### Field Experiments

Twelve seeds per genotype were sown in a 1.2 m row-plot with 20 cm of inter-row spacing at three locations (Yangzhou University farm, Shanghai farm, and Fangqiang farm) in two growing seasons (2011∼2012 and 2012∼2013). Due to the limitation of measurements of the large number of genotypes, all the DH lines and parents were sown in one row only with a random order. For all the trials, the fertilizer (Sinochem) used included: 150 kg/ha before sown, 75 kg/ha at seedling stage and 75 kg/ha at stem elongation stage. The ratio of N:P:K for the fertilizer was 7:1:2. Aphids were sprayed at both seedling and flowing stages using dimethoate (Jingjin Pesticide, Co., Ltd). Pinoxaden (Syngenta) was applied to control weeds before winter.

### Measurement of GOA

**FIGURE 1:**
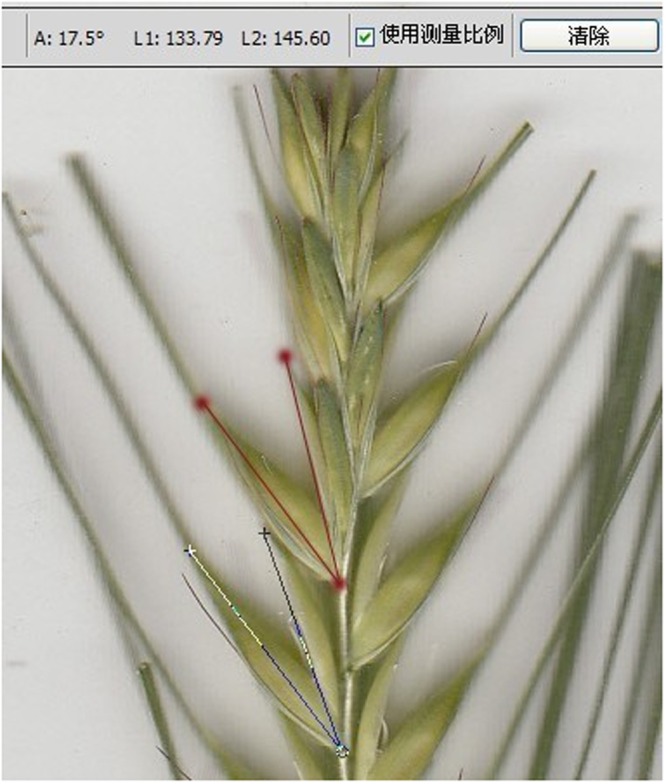
**Measurement of glume opening angle**.

Four plants of each material were randomly selected to measure GOA. Since flowering time varied among DH lines, each plant was sampled at anthesis (growth stage 65) (Zadoks et al., 1974). Panicles of the main stem and a tiller on each plant were sampled when the middle glume in the panicle opened, filaments elongated and anthers became bright yellow. The sampled panicle was cut at the reciprocal second internode and the stem was put in cold water immediately to keep the glumes opening. After sampling, lateral glumes were manually removed and the spikelet was scanned using Labscan (GE Healthcare, USA). As illustrated in **Figure 1**, GOA was measured between the inside and outside of the glume using Adobe Photoshop CS5 software. Parent Yangnongpi7 shows a glume closing phenotype with a score of 0°.

### Construction of Linkage Maps

Genomic DNA was extracted from fresh leaves of five pooled plants of each line using a simplified CTAB-based procedure modified from Murray and Thompson (1980). A total of 852 SSR (simple sequence repeats) markers were obtained from the GrainGenes database^1^. Primers with clear polymorphisms between the parents Yangnongpi7 and Yang0187 were used to genotype the DH population. PCR was conducted in 20 μL volume, consisting of 1.5 mM MgCl_2_, 0.2 uM primers, 200 uM of each dNTP, 1× PCR buffer, 50–100 ng genomic DNA and 1 U Taq DNA polymerase (TaKaRa Bio., Japan).

All markers were blasted to the consensus map (Varshney et al., 2007) and assembly_WGSMorex on IPK Barley Blast Server. A consensus map was constructed based on the genetic position of markers on the public map (Varshney et al., 2007).

### QTL Analysis

The mean GOA of main panicle and tilling panicle for each plant was used as the phenotype to analyze the QTL. Since the experiments were conducted at three locations in 2 years, QTLs, QTL × environments and QTL × QTL were analyzed by a composite interval mapping method based on a linear mixed model using the software QTLNetwork 2.0 (Yang et al., 2007). One thousand permutations were implemented and genetic effects were estimated by the Bayesian method. QTLs are named according to McCouch et al. (1997). QTLs explaining less than 10.0% of the phenotypic variation were defined as minor QTL, whereas QTL was defined as major QTL (Collard et al., 2005).

### Verification of QTLs

Referring to the published sequence of the barley cleistogamy gene *Cly1* (KJ363931) (Nair et al., 2010), *Cly1* was amplified and assembled in Yangnongpi7 and Yang0187. Blasting *Cly1* sequence between two parents, three SNPs were detected at 296, 615, and 3073 bp, respectively (Supplementary Figure S1A). Using the software Primer 3.0 (Untergasser et al., 2012), a new specific marker for barley chasmogamy gene (*cly1*), designated as KDH, was developed based on SNP (A/G) at 3073 bp (Supplementary Figure S1B). The criteria for primer designing was: primer length: 18–27 bp; melting temperature: 50–65°C; GC content: 40–60%; and polymerase chain reaction (PCR) product size: 200–400 bp. In order to validate minor QTLs, all DH lines carrying *cly1* gene were removed using KDH marker (Supplementary Figure S2). The remaining DH lines were used to re-construct genetic maps and re-map the QTLs.

### Statistical Analysis

A joint ANOVA of GOA was conducted by SPSS 16.0 using the general linear model with all sources of variation being random. Variance components and heritability of GOA were then estimated based on the mean square. Multiple comparison tests were conducted to quantify the differences of GOA among different allele combinations.

## Results

### GOA of Parents and DH Lines

The parent Yangnongpi7 showed a GOA of 0°, and the GOA of Yang0187 varied from 15.16 to 19.62° across experiments (**Table 1**). The GOA of DH lines ranged from 0 to 21.60° at three locations (**Table 1**) and distribution of GOA was multimodal (Supplementary Figure S3). Significant differences were found among genotypes and effects of location × year, year × genotype, and location × year × genotype interactions (Supplementary Table S1). Genotype contributed the most to the variation (Supplementary Table S2) with the heritability of GOA being 0.85.

**Table 1:** Glume-opening angle of two parents and 247 DH lines at three locations across two growing seasons.

Year	Site	Parents		DH lines		
		Yang0187	Yangnongpi 7	Means	Range	*CV*
2012	Yangzhou University farm	16.80°	0°	8.73 ± 5.52°	0–20.56°	63.20
	Shanghai farm	19.62°	0°	9.36 ± 6.45°	0–21.44°	68.90
	Fangqiang farm	17.68°	0°	8.83 ± 5.84°	0–19.48°	66.10
2013	Yangzhou University farm	15.16°	0°	9.47 ± 5.79°	0–21.30°	61.20
	Shanghai farm	16.54°	0°	9.22 ± 5.93°	0–20.10°	64.30
	Fangqiang farm	16.76°	0°	9.14 ± 5.52°	0–21.60°	60.40

**CV*, coefficient variation.*

### QTLs Associated with GOA

A total of 106 markers were mapped to the genetic maps covering a genome region of 898.41 cM (Supplementary Figure S4). Using this map, three QTLs associated with GOA were identified on chromosomes 2H and 7H, respectively (**Table 2**; Supplementary Figure S4). The accumulative effects of *QGOA-2H-1, QGOA-2H-2*, and *QGOA-7H* were 1.11, 4.90, and 1.48, respectively, with Yang0187 increasing GOA at three loci. Among the three QTLs, *QGOA-2H-2* at 153.8 cM on chromosome 2H was a major QTL and explained 63.92% of the phenotypic variation. The two minor QTLs on chromosomes 2H and 7H, explained 3.80 and 1.62% of the phenotypic variation, respectively. A significant effect was found with the interaction between *QGOA-2H-2* and environment, which explained 0.36% of the phenotypic variation. The interaction between *QGOA-2H-2* and *QGOA-7H* was also significant (Supplementary Table S3), which explained 0.15% of the phenotypic variation. The minor QTL *QGOA-2H-1* interacted with a locus on chromosome 6H significantly (Supplementary Figure S4), which explained 0.66% of the phenotypic variation (Supplementary Table S3).

**Table 2:** Quantitative trait loci (QTLs) identified for glume opening angle.

QTL	Interval	Position (cM)	Additive	*R*^2^_(A)_%	*R*^2^_(A__×__E)_%
*QGOA-2H-1*	Bmag0745-GBMS160	58.1	1.1089	3.8	Ns
*QGOA-2H-2*	KDH-GBM1498	153.8	4.9028	63.92	0.36
*QGOA-7H*	Bmag0206-GBS0154	16.8	1.4757	1.62	Ns

*Ns, not significant.*

### Verification of Minor QTLs

Primer sequences of the marker KDH were: forward primer 5′-TCAAACTGTGCAGTTCGTGG-3′ and reverse primer 5′-AGGGTGGGAATCGTGATAAT-3′. After removing 107 DH lines carrying *cly1* of the major QTL *QGOA-2H-2*, 140 DH lines were used to re-map QTLs associated with GOA. Only two minor QTLs *qGOA-2H-1* and *qGOA-7H* were identified (Supplementary Table S4). The percentages of the phenotypic variation determined by the two minor QTLs were 9.60 and 13.14% respectively, much higher than those identified using all DH lines (**Table 2**).

### Accumulative Effects of QTLs on GOA

Effects of the three QTLs associated with GOA were evaluated using the flanking markers. KDH was used to select lines carrying the allele of the major QTL *QGOA-2H-2*. GBMS160 and Bmag0206 were used to select lines carrying the alleles of the minor QTLs *QGOA-2H-1* and *QGOA-7H*, respectively. An average GOA of 2.6° was observed in the 24 DH lines carrying no allele of the three QTLs. This GOA of 2.6° was significantly lower than that of the lines carrying one or more alleles of the QTLs (**Table 3**). DH lines carrying the unique allele of *QGOA-2H-1* showed no significant differences of GOA to lines carrying the unique allele of *QGOA-7H*. A total of 107 DH lines carrying the allele of the major QTL *QGOA-2H-2* were chasmogamous (Supplementary Figure S5). DH lines carrying the unique allele of the major QTL showed no significant difference of GOA to those carrying the alleles of the major QTL plus one minor QTL. However, DH lines carrying the alleles of the major QTL plus two minor QTLs showed the largest GOA of 16.17° (**Table 3**; Supplementary Figure S6). Lines carrying the alleles of two minor QTLs were chasmogamous but showed a lower average GOA than those carrying the alleles of the major QTL (Supplementary Figure S7).

**Table 3:** Glume opening angle of DH lines with different QTLs.

No.	Alleles combination^∗^	Number of lines	Mean	Significance^∗∗^
1	None QTL	24	2.60 ± 0.70°	e
2	*mi2H*	29	5.04 ± 0.71°	d
3	*mi7H*	40	4.72 ± 0.57°	d
4	*mi2H+ mi7H*	45	6.87 ± 0.46°	c
5	*ma2H*	15	13.77 ± 0.65°	b
6	*ma2H+ mi2H*	30	14.30 ± 0.58°	b
7	*ma2H+ mi7H*	29	13.38 ± 0.27°	b
8	*ma2H+mi2H+mi7H*	29	16.17 ± 0.26°	a

*^∗^*mi2H* stands for the allele of the minor QTL QGOA-2H-1. *mi7H* stands for the allele of the minor QTL *QGOA-7H*. *ma2H* stands for the allele of the major QTL *QGOA-2H-2.*^∗∗^*P* ≤ 0.05. a: lines carrying the *ma2H+mi2H+mi7H* alleles had significantly larger GOA than lines carrying the other alleles combinations. b: lines carrying either *ma2H+ mi7H, ma2H+ mi2H, or ma2H* showed no difference in their GOA, but the GOA was higher than lines carrying alleles combination 1, 2, 3, and 4. c: lines carrying *mi2H+ mi7H* was higher than lines carrying alleles combination 1, 2, and 3. d: lines carrying either *mi2H or mi7H* showed no difference in their GOA, but the GOA was higher than lines carrying alleles combination 1. e: lines carrying no allele had significantly lower GOA than lines carrying alleles of one or more QTLs.*

## Discussion

Self-pollination not only protects crops against diseases spread by flower tissue, but also keeps seeds pure. However, utilization of heterosis is limited in many self-pollination crops. For barley, the fourth major cereal crop in the world, very limited progress has been made since the first barley genetic male sterile (GMS) line was reported in Suneson (1940). One of the key issues is that the glume of the barley is closed during flowering.

Cleistogamous and chasmogamous are two opposing phenomena for barley during flowering. Cleistogamous and chasmogamous can be assessed visually. Plants with emerged anthers from florets during the pollination phase were scored as chasmogamous (Turuspekov et al., 2004, 2009). Cleistogamy and chasmogamy are difficult to measure under field conditions, alternative methods were used to measure cleistogamy and chasmogamy. The size of barley lodicules was used as phenotype and barley cleistogamy gene *Cly1* was detected successfully (Nair et al., 2010). In our previous studies (Lv et al., 2009; Lan et al., 2013), a new method was developed to measure cleistogamy and chasmogamy by GOA. GOA can be measured using a scanner and the software Photoshop CS5. The method described in this paper provided an effective approach for studying glume opening traits in crops.

Nair et al. (2010) regarded cleistogamy and chasmogamy as qualitative characters, and our studies used GOA to quantify cleistogamy and chasmogamy in different populations (current study and Lv et al., 2009). The previous study (Lv et al., 2009) showed that both a major gene and multiple minor genes controlled the GOA. In this study, a total of five QTLs were identified, and three of them could be identified at all environments. The major QTL *QGOA-2H-2* explained 63.92% of the phenotypic variation with flanking markers of KDH and GBM1498. GBM1498 was also the closest marker for the genes *cly1* and *Cly2* on chromosome 2HL (Turuspekov et al., 2004). Therefore, the major QTL is co-located with *cly1*, which is a major gene determining cleistogamy and chasmogamy. Since cleistogamy and chasmogamy were difficult to measure under field conditions, a functional marker KDH for GOA was developed based on the sequence differences of *cly1* gene. This marker could identify most of the chasmogamous DH lines, which could be successfully used in barley breeding programs.

Two minor QTLs for GOA, *qGOA-2H-1* and *qGOA-7H*, were identified and confirmed using the lines with no major chasmogamy gene *cly1*. Aligning the position of QTLs with rice chromosomes by flanking markers, *qGOA-2H-1* was in synteny with rice chromosome 7 from 89.6 to 94.4 cM (Supplementary Figure S8A). In this interval, no glume opening related genes were found. The gene *OsMADS18* was close to this synteny chromosome, which was a transcription factor for the development of flower (Becker and Theissen, 2003). For the minor QTL *qGOA-7H*, two rice syntenic intervals were found on chromosomes 6 and 8, respectively (Supplementary Figure S8B). On rice chromosome 8, a QTL associated with maximal GOA was mapped with flanking markers of RZ143 and C400a (Uga et al., 2003). However, this QTL was not within the syntenic interval. To our knowledge, the two minor QTLs identified in this study were first reported in barley.

## Author Contributions

XZ and BG measured the GOA, performed SSR assays, analyzed the QTLs, developed the KDH marker and wrote the manuscript. HL and GL determined the GOA and prepared the DNA for SSR assays. SL and JM determined the GOA and performed the PCR to validate the effects of minor QTLs. CL managed the field experiments. RX supervised the project.

## Conflict of Interest Statement

The authors declare that the research was conducted in the absence of any commercial or financial relationships that could be construed as a potential conflict of interest.

## Footnotes

1 http://wheat.pw.usda.gov/GG3/
