# Supplementary material for: A Major QTL, Which Is Co-located with cly1, and Two Minor QTLs Are Associated with Glume Opening Angle in Barley (Hordeum vulgare L.)
Source: Front Plant Sci. 2016 Oct 24;7:1585. doi: 10.3389/fpls.2016.01585 (PMC5075565; doi:10.3389/fpls.2016.01585)
Supplement: Supplementary file 1 [file Presentation_1.pdf]

Supplementary Material

A major QTL, which is co-located with *cly1*, and two minor QTLs are associated with glume opening angle in barley (*Hordeum vulgare* L.)

XinZhong Zhang¶, BaoJian Guo¶, GuoFang Lan, HongTao Li, Shen Lin, Jun Ma, Chao Lv, RuGen Xu*

*** Correspondence:** Dr. Rugen Xu: rgxu@yzu.edu.cn

1 Supplementary Data

Supplementary Material should be uploaded separately on submission. Please include any supplementary data, figures and/or tables.

Supplementary material is not typeset so please ensure that all information is clearly presented, the appropriate caption is included in the file and not in the manuscript, and that the style conforms to the rest of the article.

2 Supplementary Figures and Tables

For more information on Supplementary Material and for details on the different file types accepted, please see here.

2.1 Supplementary Figures

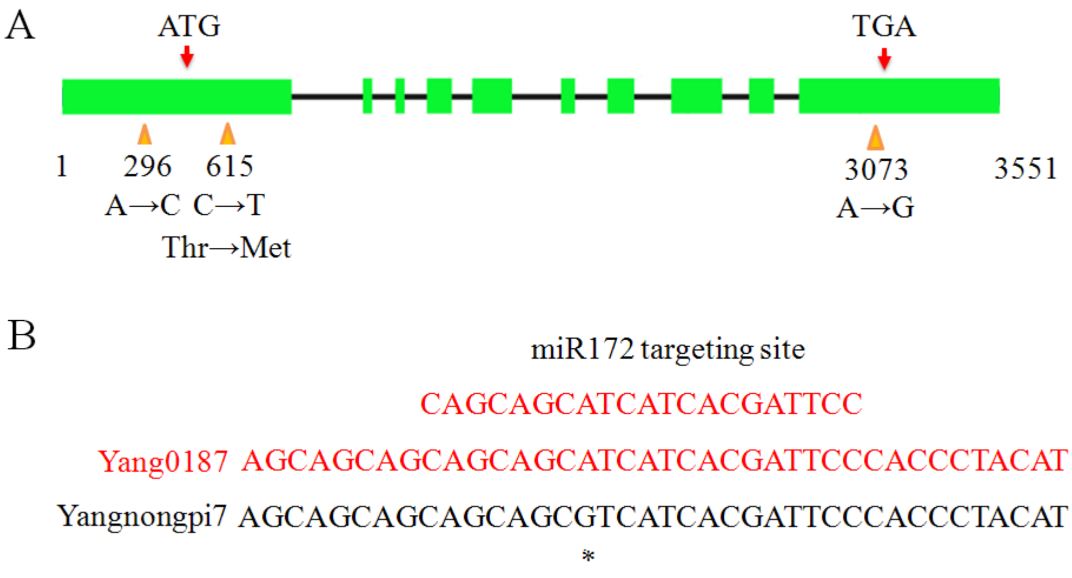

Figure S1. Gene structure and SNP of *Cly1* gene between Yangnongpi7 and Yang0187. (A) Gene structure and SNPs of *Cly1*, green boxes and black line indicated the exon and intron, respectively. (B) Sequence comparisons between Yangnongpi7 and Yang0187 at a putative miR172 target site. Black star indicated the SNP site.

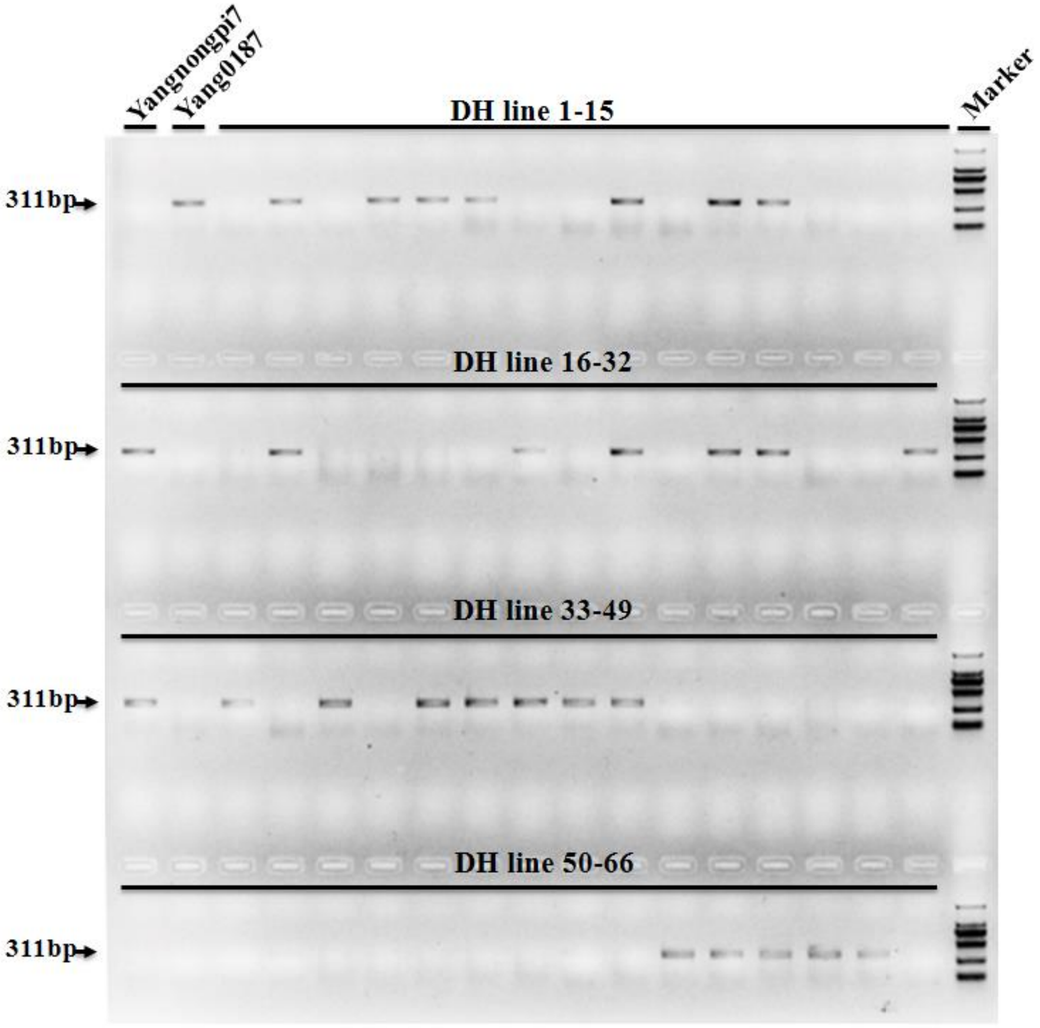

Figure S2. Selecting of DH lines carrying *cly1* using the specific marker KDH.

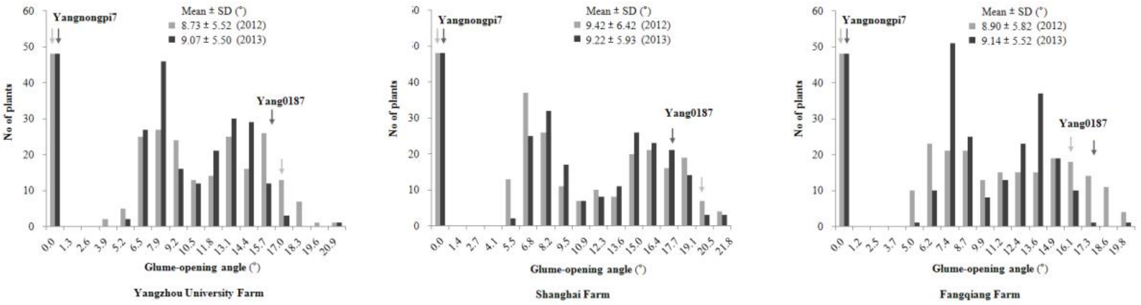

Figure S3. Frequency distributions of glume opening angle in the DH lines derived from a cross Yangnongpi7 and Yang0187.

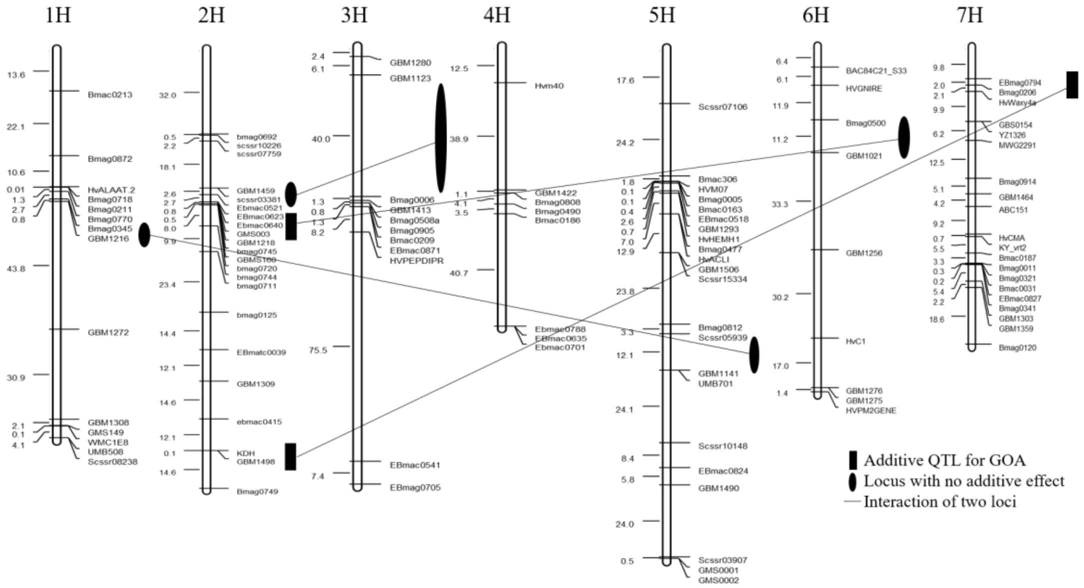

Figure S4. Additive and epistasis effects detected for barley glume opening angle.

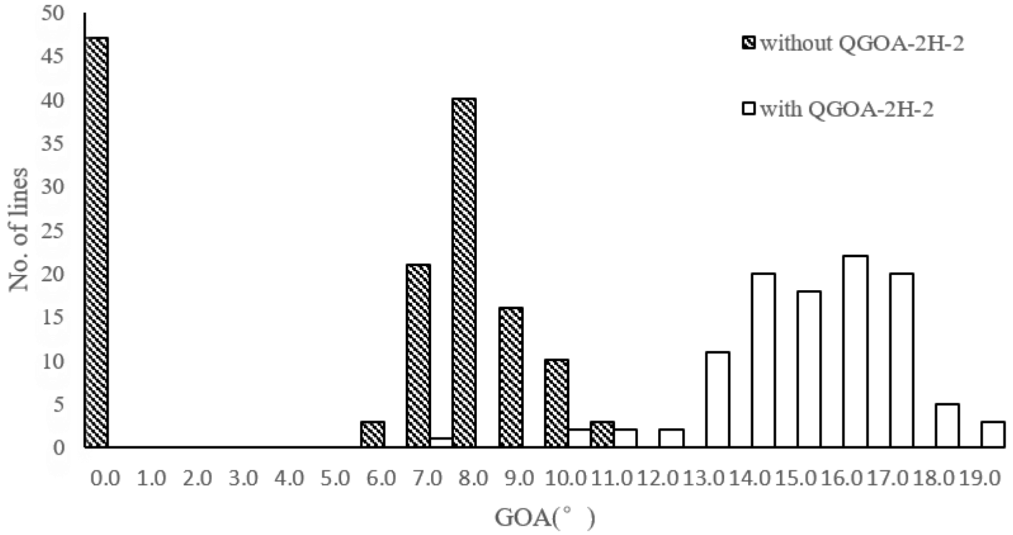

Figure S5. Distribution of glume opening angle for DH lines with and without the allele of the major QTL *QGOA-2H-2*.

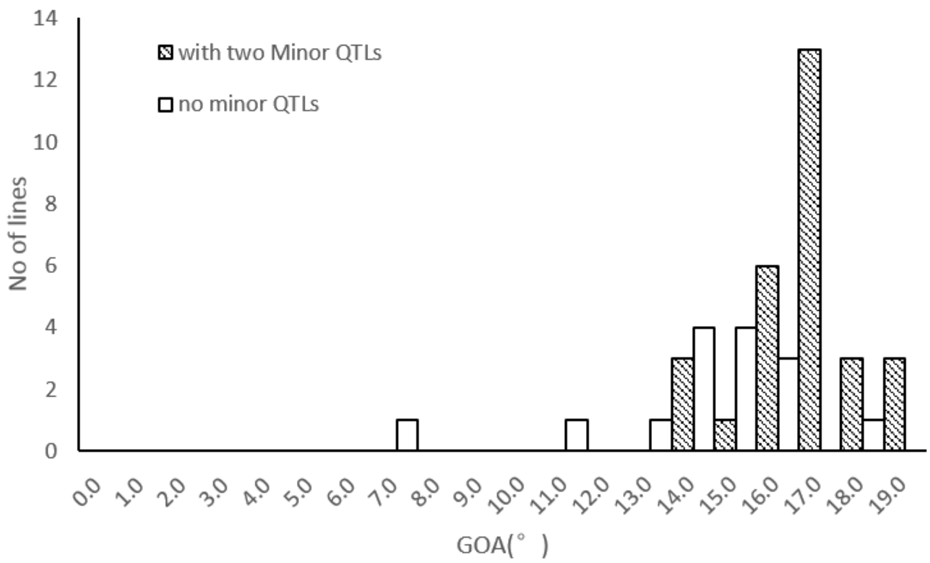

Figure S6. Distribution of glume opening angle for cleistogamous DH lines with and without the alleles of the minor QTLs

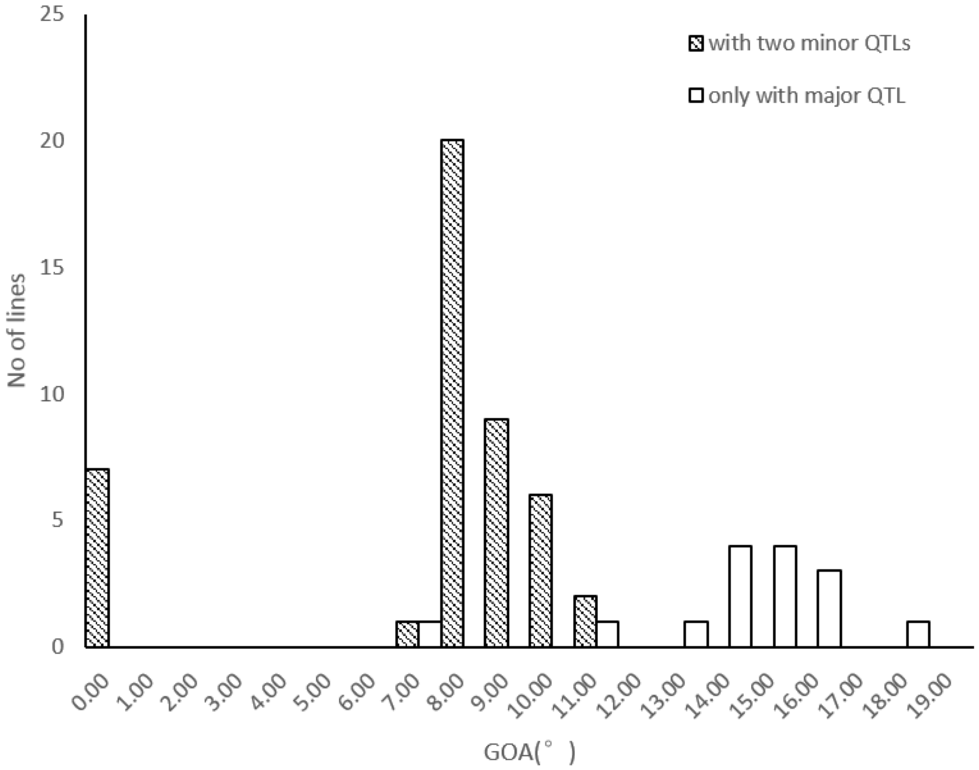

Figure S7. Distribution of GOA for DH lines carrying the alleles of the the two minor QTLs and lines carry the unique allele of the major QTL.

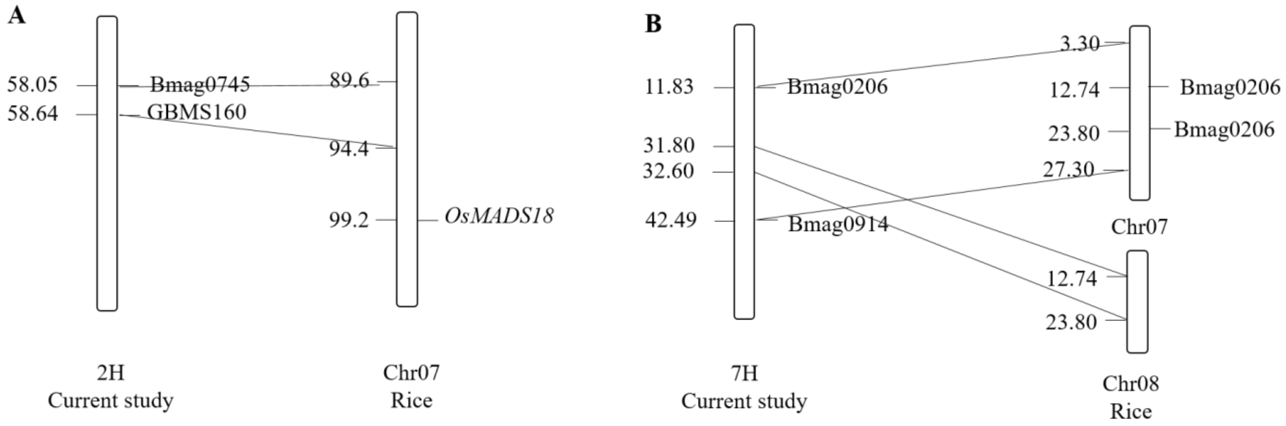

Figure S8. Aligning the position of QTLs with rice chromosomes by flanking markers.

2.2 Supplementary Tables

Table S1 ANOVA of glume opening angle in DH population

S.O.V.	*df*	*SS*	*MS*	*F*
Location	2	1146.26	573.13	0.45
Year	1	147.06	147.06	0.11
Genotype	246	177898.71	726.165	23.23**
Location*Year	2	2571.52	1285.76	83.13**
Location×Genotype	492	6744.15	13.71	0.89
Year×Genotype	246	7658.07	31.13	2.01**
Location×Year×Genotype	492	7609.64	15.47	2.99**
Error	4446	22991.07	5.17	

**Significant at p≤ 0.01

Table S2 Variance components and heritability of glume opening angle

V_E	$V_{L×Y×G}$	$V_{G×Y}$	$V_{L×Y}$	V_G	h^2
5.17	2.58	1.31	1.29	28.91	0.849

Table S3 Epistasis effects of QTLs for glume opening angle

QTL1	Interval	QTL2	Interval	A×A	R^2 %
QGOA-2H-2	KDH-GBM1498	*QGOA-7H*	Bmag0206-GBS0154	-1.0197	0.15%
QGOA-1H	Bmag0345-GBM1216	*QGOA-5H-1*	Scssr05939-gbm1141	0.3591	1.02%
QGOA-2H-1	Bmag0745-GBMS160	*QGOA-6H-1*	Bmag0500-GBM1021	-0.5088	0.66%
QGOA-2H-3	EBmac0521-Ebmac0623	*QGOA-3H*	GBM1123-Bmag0006	-0.4999	0.48%

Table S4 Additive effect of the two minor QTL for glume opening angle after removing the DH lines carrying *cly1*

QTL	Interval	Position (cM)	Additive	R^2 (A)%
QGOA-2H-1	Bmag0745-GBMS160	58.1	1.5608	9.60%
QGOA-7H	Bmag0206-GBS0154	16.8	2.2030	13.14%
